# Combinatorial treatment with topical NSAIDs and anti-VEGF for age-related macular degeneration, a meta-analysis

**DOI:** 10.1371/journal.pone.0184998

**Published:** 2017-10-06

**Authors:** Songshan Li, Andina Hu, Wei Wang, Xiaoyan Ding, Lin Lu

**Affiliations:** State Key Laboratory of Ophthalmology, Retina Division, Zhongshan Ophthalmic Center, Sun Yat-sen University, Guangzhou, Guangdong, China; University of Tennessee Health Science Center, UNITED STATES

## Abstract

Inflammation is a key pathogenic factor in age-related macular degeneration (AMD). However, the clinical importance of combining anti-VEGF agents and topical NSAIDs to reduce inflammation remains unclear. In this study, we systematically reviewed clinical trials comparing combined treatment versus anti-VEGF alone in AMD patients. We quantified treatment effects via meta-analysis. The pooled weighted mean difference (WMD, -0.91, 95%CI: -1.39 to -0.42, P = 0.0003) demonstrates that combined treatment may reduce required anti-VEGF injection number, probably by means of decreasing central retina thickness (CRT) (WMD = -22.9, 95% CI: -41.20 to -4.59, P = 0.01). The best corrected visual acuity (BCVA) did not change significantly between these two groups (WMD = - 0.01, 95%CI: -0.23 to 0.20, P = 0.90). Topical NSAIDs slightly increased the incidence of foreign body sensation (Odds Ratio [OR] = 2.63, 95%Cl: 1.06 to 6.52, P = 0.76). Combining topical NSAIDs and anti-VEGF agents may provide a new strategy for AMD treatment.

## Introduction

Age-related macular degeneration (AMD) is the third leading cause of blindness worldwide, after cataracts and glaucoma [1] The central vision lost as a result of AMD is irreversible and permanent. AMD produces suffering for the patient and presents a heavy financial burden both to the family of the patient and to society as well. Various factors are involved in the pathogenesis of neovascular AMD (nAMD, an AMD subtype), including aging, oxidative stress, genetic factors, inflammation, and increased vascular endothelial growth factor (VEGF). It appears that VEGF is important in the pathogenesis of AMD. Anti-VEGF treatments, including Ranibizumab, Bevacizumab, and Aflibercept, have shown significant benefit in AMD.

However, some patients respond slowly or poorly to anti-VEGF treatment, suggesting that other pathogenic factors should also be considered as treatment targets. Since various studies demonstrate the role of inflammation in the pathogenesis of neovascularization, it is compelling to consider anti-inflammatories as adjunctive therapy [2][3][4]. Unlike intraocular steroids, which carry with them substantial side-effects [5], topical nonsteroidal anti-inflammatory agents (NSAIDs) offer simple, safe, and effective means to reduce inflammation through prevention of prostaglandin synthesis via cyclooxygenase inhibition.

There has been considerable evidence for the effectiveness of topical NSAIDs in preventing cystoid macular edema after cataract surgery [6]. However, the literature is mixed regarding the effect of NSAIDs in nAMD. Several studies have compared the number of anti-VEGF intravitreal injections needed, best corrected visual acuity (BCVA), central retina thickness (CRT) and side effects following anti-VEGF treatment combined with or without NSAIDs [7][8][9][10]. However, the sample sizes were small, casting doubt on the validity of the studies. Here, we perform a meta-analysis of clinical trials, summarizing the effects of combining topical NSAIDs and anti-VEGF in nAMD patients.

## Methods

This study was performed according PRISMA checklist guidelines, and is registered in PROSPERO, number CRD42016039935.

### Literature search

A systematic literature review was performed to identify relevant articles comparing anti-VEGF agents combined with topical NSAIDS and anti-VEGF alone for the treatment of nAMD from inception to December 2016. Two independent reviewers (SL, AH), searched electronic databases including PubMed, EMBASE and the Cochrane Central Register of Controlled Trials. Following key words were used: “anti-VEGF OR ranibizumab OR bevacizumab OR Aflibercept OR Lucentis OR Avastin OR Eylea” AND “macular degeneration OR AMD OR ARMD” AND “anti-inflammatory agents non-steroidal OR NSAIDs OR bromfenac OR diclofenac OR ketorolac OR nepafenac”. No language restrictions were set for the search. References of included studies and major reviews were searched manually for additional eligible studies. Grey articles were searched from the OpenGrey website and Google Scholar. After excluding duplications derived from different sources, article titles and abstracts were evaluated by two independent researchers to exclude case reports, cross-sectional studies, retrospective studies and unrelated articles. The full texts of remaining articles were examined for eligibility. Disagreements were settled by consensus or a third senior reviewer (XD) if needed.

The following including criteria were used to check study eligibility: (1) Design: prospective RCT or quasi-RCT; (2) Patients: treated or naïve wet AMD requiring anti-VEGF therapy; (3) Intervention and control: anti-VEGF combined with topical NSAIDS (study group) versus anti-VEGF alone (control group); (4) Outcomes: at least one of the following: injection number of anti-VEGF, best corrected visual acuity (BCVA), and central retinal thickness (CRT); (5) Other: a minimum follow-up of 6 months. Studies were excluded if any of the following conditions existed: (1) retrospective cohort study, case control study, or cross sectional study; (2) studies with inadequate information for calculating data on outcomes; (3) duplicated report; (4) study with a sample size smaller than 10 eyes in each arm; (6) mean observation period shorter than 6 months. If multiple publications on the overlapping population were available, the most comprehensive one was included in the meta-analysis. If multiple studies by the same team derived from different patients were available, all of them were deemed eligible.

### Data extraction

Data were extracted from each study by two independent reviewers (SL, AH) with a standardized form, including first author name, publication time, patient and study characteristics, follow-up duration, sample size, type of anti-VEGF, method of anti-VEGF treatment, type of NSAID, method of NSAID treatment, injection number of anti-VEGF, BCVA at the end point, CRT at the end point, number of patients with adverse effects of NSAID treatment. All BCVA values were transferred into the log minimum angle of resolution (LogMAR) for analysis. Additional data and information were requested and obtained from authors through email contacts if data or information were not explicitly presented in the articles. Any discrepancies in data extraction were resolved via re-assessment and discussion with the third reviewer (XD).

### Evaluation of risk of bias

Risk of bias of each included study was evaluated using the Cochrane risk of bias tool, as described in the Cochrane systematic review of interventions manuals. This tool evaluated the risk of bias in seven domains: random sequence generation, allocation concealment, blinding of participants and personnel, blinding of outcome assessment, incomplete outcome data, selective reporting, and other sources of bias. The risk of bias in each domain was labeled as low, unclear or high risk for each study by two authors (SL and AH) independently. Disagreements were resolved as described above.

### Statistical analysis

Meta-analysis was performed using RevMan (version5.2.3). Weight mean differences (WMD) were used for continuous variables, and odds ratios (OR) were used for dichotomous variables. Pooling estimates and their 95% confidential intervals (95%CI) were calculated. The random-effects model was used to calculate pooling estimates when significant inter-study heterogeneity existed. Otherwise, the fixed-effects model was adopted. Forest plots were produced for a clearer visualization. Statistical heterogeneity among studies was examined using the Cochran Q test, with a P < 0.10 indicating significant heterogeneity. Heterogeneity among studies also was quantified using the I^2^ statistic, which represents the percentage of the total variation among studies. An I^2^ value of 25%-50%, 50%-75%, > 75% was defined as low, moderate, and severe inter-study heterogeneity, respectively. Analyses were stratified by study design, types of topical NSAIDs, types of anti-VEGF agents, and follow-up time. One-way sensitivity analyses were performed by recombining the studies after deleting one study at a time sequentially. Potential publication bias was assessed by examining the symmetry of funnel plots and Egger’s test if more than 10 studies were included.

## Results

### Literature search

**Fig 1:**
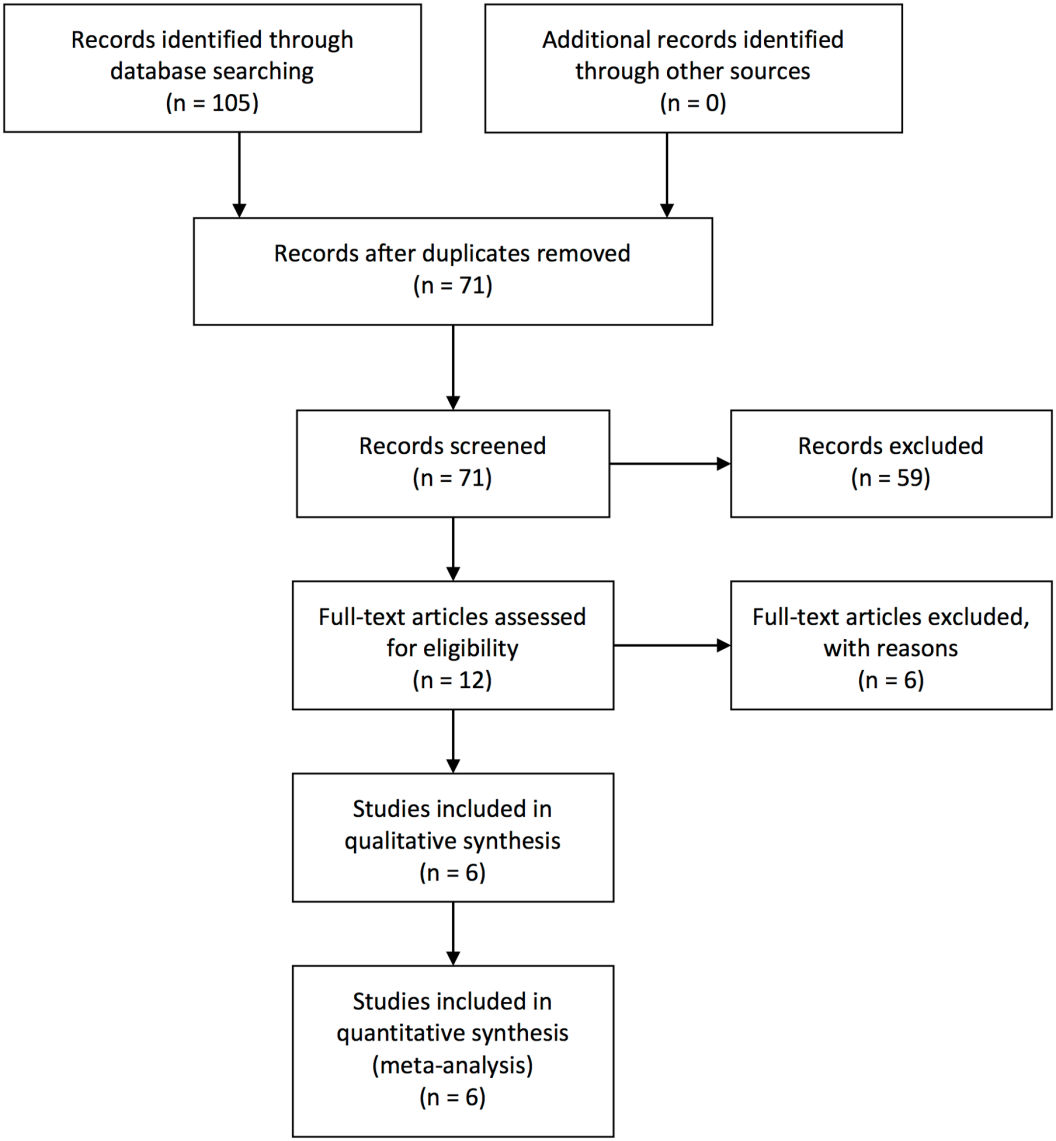
Flow diagram for literature searching and inclusion.

The search process is illustrated as a flow diagram in Fig 1. Initially, a total of 105 articles were retrieved from databases. No related grey articles were found. After removing 34 duplicates, the titles and abstracts of 71 potential relevant articles were reviewed. Among the 71 papers, 59 were excluded because of un-relevant topics. Full texts of the remaining 12 publications were assessed in their entirety. Six were excluded as they were case reports or case series (two), editorials or comments (one), or retrospective studies (three). The remaining six studies, including two quasi-RCTs and four RCTs, were included in this meta-analysis.

### Characteristics of included studies

Details of the features of included studies are shown in Table 1. Four RCTs and two quasi-RCTs were included in the mata-analysis, involving a total of 278 patients (142 in study group and 136 in control group). All studies were published between 2012 and 2015. Two studies were conducted in Italy, two in Poland, and the other two in the United States and Japan, respectively. Each study compared the BCVA and/or central retinal thickness (CRT) folloiwng combined treatment with topical NSAIDs and anti-VEGF or anti-VEGF alone in nAMD patients. Five papers reported the injection number of anti-VEGF needed with or without topical NSAIDs. One of these did not provide the standard deviation and was therefore excluded meta-analysis. Follow up duration for all studies were between 6 months to 12 months. Of the 278 eyes, 172 (62%) in four trials received ranibizumab as the anti-VEGF agent, 54 eyes (19%) in one trial received aflibercept, and 52 eyes (19%) in another trial received bevacizumab as anti-VEGF agent. Three Studies looked at treatment with anti-VEGF using the 3 + PRN protocol, two studies used the 4 + PRN protocol and one used the 1 + PRN protocol. Bromfenac was used in four studies, including 89 eyes (63%). Ketorolac was employed in two trials, including 53 eyes (37%). Adverse effects of topical NSAIDs were assessed in three trials.

**Table 1:** Baseline characteristics of the included studies.

Study	Design	Country	Patients	No. of patients	Mean age	Anti-VEGF	NSAIDs	Follow-up	Outcomes
Flaxel et al., 2012[10]	RCT	United States	New or recurrent exudative or neovascular AMD	20/10	85.5/77.5	Ranibizumab (4+PRN)	Bromfenac (1 drop twice daily for 12 months)	12	BCVA, CRT
Gomi et al., 2012[8]	RCT	Japan	nAMD with lesions smaller than 2 disk diameters	16/22	75/74.4	Ranibizumab (1+PRN)	Bromfenac (1 drop twice daily for 6 months)	6	CVA, CRT, No.of injection
Russo et al., 2013[11]	RCT	Italy	New neovascular AMD	28/26	76/77.8	Ranibizumab (3+PRN)	Ketorolac (1 drop three times a day for 6 months)	6	CVA, CRT, No.of injection
Wyględowska-Promieńska et al., 2014[12]	Quasi-RCT	Poland	Exudative AMD	26/26	72.4/72.3	Bevacizumab (3+PRN)	Bromfenac (1 drop twice daily for 3 months)	8	CVA, CRT, No.of injection
Semeraro et al., 2015[13]	RCT	Italy	Naïve eyes affected by neovascular AMD	25/25	76.3/77.2	Ranibizumab (3+PRN)	Ketorolac (1 drop three times a day for 12 months)	12	CVA, CRT, No.of injection
Wyględowska-Promieńska et al., 2015[14]	Quasi-RCT	Poland	Exudative AMD	27/27	72.3/72.8	Aflibercept (4+PRN)	Bromfenac (1 drop twice daily for 3 months)	8	BCVA, CRT

### Risk of bias of the included studies

Study quality was assessed using the Cochrane Risk of Bias Tool for RCTs, which classify the involved trials to low risk, unclear risk and high risk (Table 2).

**Table 2:** Assessment of risk of bias of the included studies.

Domain	Flaxel et al. 2012	Gomi et al. 2012	Russo et al. 2013	Wyględowska-Promieńska et al., 2014	Semeraro et al. 2015	Wyględowska-Promieńska et al. 2015
Random sequence generation	Low risk	Low risk	Low risk	High risk	Low risk	High risk
Allocation concealment	Unclear	Unclear	Unclear	High risk	Unclear	High risk
**Blinding for visual acuity**						
Participants and personnel	High risk	Low risk	High risk	High risk	High risk	High risk
Outcome assessment	Low risk	Low risk	Low risk	Low risk	Low risk	Low risk
**Blinding for other outcomes**						
Participants and personnel	High risk	Low risk	High risk	High risk	High risk	High risk
Outcome assessment	Low risk	Low risk	Low risk	Low risk	Low risk	Low risk
Incomplete outcome data	Low risk	Low risk	Low risk	Low risk	Low risk	Low risk
Selective reporting	Low risk	Low risk	Low risk	Low risk	Low risk	Low risk
Other bias	Low risk	Low risk	Low risk	Low risk	Low risk	Low risk

### Results of meta-analysis

**Fig 2:**
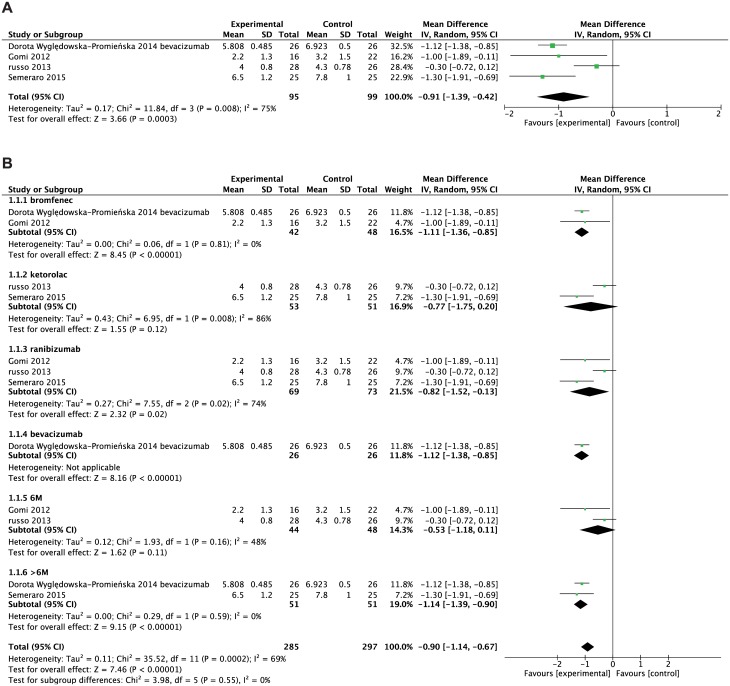
Forest plot showing the weighted mean difference of required anti-VEGF injections, comparing combined treatment and anti-VEGF alone. A. Pooled data computed using the random effects model. B. Data was grouped by type of NSAIDs (bromfenac and ketorolac), type of anti-VEGF (ranibizumab and bevacizumab) and follow-up duration (6 months and greater than 6 months).

Four studies compared the mean injection numbers between treatment and control group. Pooling results showed that combined topical NSAIDs with anti-VEGF was associated with fewer anti-VEGF injections (WMD = -0.91, 95%CI: -1.39 to -0.42, P = 0.0003, Fig 2A). Because severe heterogeneity among studies was observed (P = 0.008, I^2^ = 75%), the random effect model was adopted. Subgroup studies were assessed according to type of topical NSAID, anti-VEGF, and duration of follow-up (Fig 2B). Regardless of anti-VEGF agent used, combined treatment decreased the number of anti-VEGF treatments required. This trend is more significant with follow-up duration greater than 6 months. However, only bromfenec demonstrated a statistically-significant reduction of anti-VEGF injection number.

**Fig 3:**
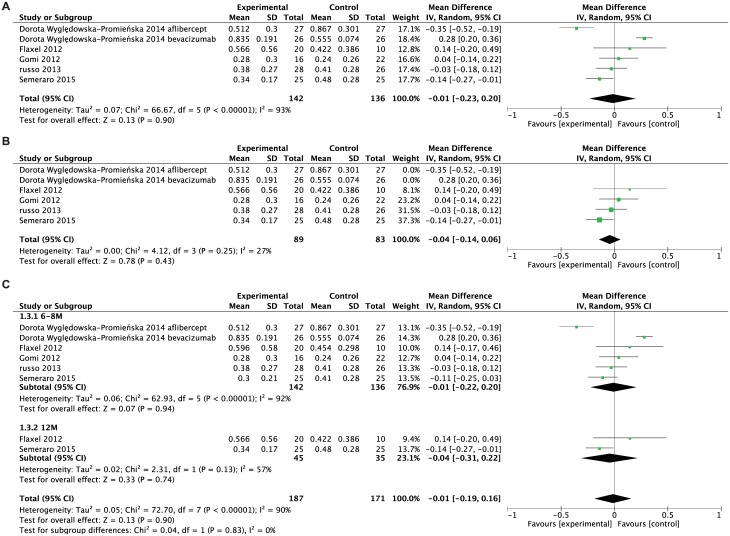
Forest plot showing weighted mean difference of BCVA comparing combined treatment and anti-VEGF alone. A. Pooled data computed the BCVA at the end point between study and control group by using the random effects model. B. Pooled data showed the results did not change after removing two quasi-RCTs. C. Data was grouped by follow-up duration (6–8 months and 12 months).

To evaluate the clinical treatment effect of the combined strategy, we analyzed the possibility of improving visual acuity in these two groups. The mean BCVA (logMAR) at final follow-up in the combined treatment group and anti-VEGF alone group were not statistically significant (Fig 3A), with the WMD = - 0.01, 95%CI: -0.23 to 0.20, P = 0.90. Since we detected severe heterogeneities among studies (P < 0.00001, I^2^ = 93%), a subgroup analysis was performed to identify the source of heterogeneity. The BCVAs from two quasi-RCTs were strongly different from other studies in the forest plot. Therefore, the two quasi-RCTs were excluded from the analysis owing to differences in study design. After removing quasi-RCTs, the heterogeneity decreased but yet failed to detect significant change (P = 0.25, I^2^ = 27%, Fig 3B). A grouping was also examined with respect to follow-up duration. This also failed to show any difference between the two groups (Fig 3C).

**Fig 4:**
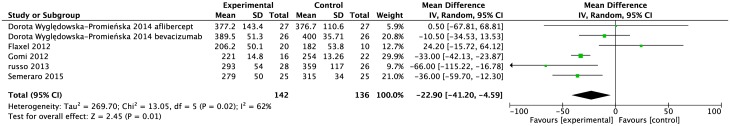
Forest plot showing weighted mean difference of CRT comparing combined treatment and anti-VEGF alone using the random effects model.

In addition to BCVA, CRT is crucial to assessment of treatment effectiveness. Fig 4 shows mean CRT at final follow-up in study group and control group. Combining topical NSAIDs with anti-VEGF may reduce the CRT significantly (followed up from 6 months to 12 months), with WMD of -22.9, 95% CI: -41.20 to -4.59, P = 0.01. Heterogeneity between studies was 62% (I^2^). Therefore, the random effect model was used.

**Fig 5:**
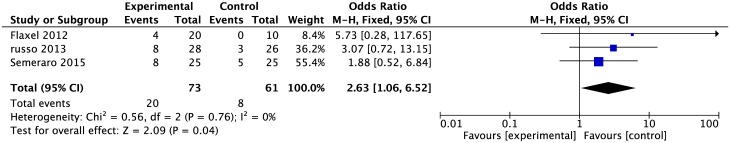
Forest plot showing the odds ratio of foreign body sensation after combined treatment or anti-VEGF alone using a fixed-effects model.

The adverse effects of topical NSAIDs in both groups were analyzed. Common adverse effects include abnormal sensation, stinging, pain, itchy eye, headache, sore eyelid, foreign body sensation, viral conjunctivitis, dry eye, eye strain, light sensitivity and floaters. Of these, only foreign body sensation significantly increased with topical NSAIDs (odds ratio [OR] = 2.63, 95%Cl: 1.06 to 6.52, P = 0.76, Fig 5). Heterogeneity between studies was 0% (I^2^) in a fixed-effects model. Pooled estimates of other adverse effects are summarized in Table 3.

**Table 3:** Pooling estimates of adverse effect of NSAIDs.

Side effect	OR	95%Cl	P	I^2^(Fixed)	Studies
Abnormal sensation	1.01	0.41–2.46	0.95	0%	3
Stinging	1.90	0.93–3.88	0.90	0%	3
Pain	0.67	0.27–1.70	0.67	0%	3
Itchy eye	1.00	0.45–2.20	0.46	0%	3
Headache	0.86	0.38–1.93	0.78	0%	3
Sore eyelid	0.68	0.18–2.53	0.82	0%	3
Foreign body sensation	2.63	1.06–6.52	0.76	0%	3
Viral conjunctivitis	0.31	0.06–1.66	0.86	0%	3
Dry eye	2.84	0.12–64.87	0.93	0%	3
Eye strain	1.37	0.56–3.36	0.97	0%	3
Light sensitivity	0.64	0.29–1.45	0.58	0%	3
Floaters	0.90	0.43–1.90	0.50	0%	3

A sensitivity analysis was performed by omitting one study at a time and re-examining remaining data. No study had a significant effect on pooled data (not shown). As only six studies were included in the meta-analysis, potential publication bias was not examined.

## Discussion

Anti-VEGF is currently the first line therapy for nAMD. Recently, however, increasing attention is being paid to alternative strategies, including combination with anti-inflammatory agents. Topical NSAIDs can achieve effective concentrations in the vitreous body with limited side-effects [15]. Although conflicting results have been reported in early retrospective studies, recent clinical trials demonstrated a beneficial trend of combining topical NSAIDs and anti-VEGF compared with anti-VEGF alone. Because of small sample sizes and mild measured effects, these studies failed to demonstrate statistical significance. Prior to carrying out a large-scale clinical trial, a meta-analysis pooling data from multiple studies may provide important insights into combination therapy.

In our meta-analysis, we included four well-designed RCTs and two quasi-RCTs, focusing on number of anti-VEGF treatment required, BCVA, CRT following treatment, and side-effects of topical NSAIDs. We found that addition of topical NSAIDs leads to a mild but stable reduction in the number of required anti-VEGF injections. This suggests use of NSAIDs may strengthen or maintain the effect of anti-VEGF. This effect is more pronounced at one-year follow up. Though topical NSAIDs show no effect on BCVA, we clearly observed a benefit on decreasing central retinal thickness. This suggests that topical NSAIDs may aid in decreasing retinal edema and sub-retinal fluid absorption. Only bromfenec but not ketorolac demonstrated a statistically-significant reduction of anti-VEGF injection number. More clinical trials may need to conclude the effect of ketorolac in decreasing the required anti-VEGF treatment. Mechanism of bromfenec and ketorolac are slightly different. Bromfenac is more selective to COX-2 while ketorolac being more selective to COX-1, which may be associated with the different therapeutic effects. [16].

A key strength of this meta-analysis is that all including studies are clinical trials. Although some are pilot studies, all are well-designed. However, several limitations should be taken in account. First, all included studies have small number of participants, lowering the power of the analysis. Nevertheless, the evidence level of this meta-analysis is higher compared with single studies. Second, the number of included studies is too small to carry out publication bias testing. In the study, related unpublished results were not found. An update of this meta-analysis may be necessary as further RCTs are published. Two of the included studies are quasi-RCTs, which do not have a trusted randomization process. In this analysis, we used a sensitivity test and found that excluding any study did not affect the final result. Finally, follow-up duration of all included studies is up to one year. Longer term studies are needed to establish the effect of sustained combination treatment.

## Conclusion

Combining topical NSAIDs with intravitreal anti-VEGF results in a small but statistically significant reduction in required anti-VEGF injections and central retinal thickness. BCVA was not improved significantly. No additional side effects were observed apart from foreign body sensation. Combining topical NSAIDs and anti-VEGF agents may serve as a new strategy in AMD treatment. Conclusions drawn from this meta-analysis should be interpreted cautiously owing to the limited number of participants in included studies.

## Supporting information

S1 Table PRISMA checklist. (DOC)
